# Tyrosine hydroxylase deficiency—Clinical insights and a novel deletion in *TH* gene in an Indian patient

**DOI:** 10.1002/jmd2.12111

**Published:** 2020-02-29

**Authors:** Sunita Bijarnia‐Mahay, Vivek Jain, Beat Thöny

**Affiliations:** ^1^ Institute of Medical Genetics & Genomics Sir Ganga Ram Hospital New Delhi India; ^2^ Department of Pediatrics and Pediatric Neurology Santokba Durlabhji Memorial Hospital Jaipur India; ^3^ Division of Metabolism University Children's Hospital Zurich Zürich Switzerland

**Keywords:** hypokinesia, infantile parkinsonism, oculogyria, tremor, tyrosine hydroxylase deficiency

## Abstract

Tyrosine hydroxylase deficiency is a rare autosomal recessive, treatable disorder of neurotransmission. Fewer than 100 cases have been reported so far. We present a case of a 10‐month‐old infant who was symptomatic since 5 months of age and who received an initial diagnosis of infantile tremor syndrome. She presented with rest tremor, decreased facial expression, global hypokinesia, and later on with oculogyric crisis and dystonia. This diagnosis was revised after confirmation of tyrosine hydroxylase deficiency by CSF neurotransmitter analysis. Genetic studies revealed one previously reported missense variant, p.Thr399Met, and another large deletion starting upstream of exon 1 and encompassing exon 1. She was started on treatment with escalating doses of L‐Dopa/Carbidopa, with folinic acid supplementation. At 3.5 years of age, her cognitive functioning and development is appropriate for age. There is complete subsidence of dystonia and oculogyric episodes. She has occasional chorieform movements which appear to be drug related.

**SYNOPSIS**

Early diagnosis even of a severe TH deficiency leads to better outcome.

## 1 INTRODUCTION

Tyrosine hydroxylase (TH) deficiency (OMIM ♯605407; encoded by the *TH* gene OMIM *191290) is an autosomal recessive disorder of L‐Dopa biosynthesis, the rate‐limiting step for the generation of the catecholamine neurotransmitters dopamine, norepinephrine, and epinephrine.^1^ This monoamine neurotransmitter disorder, also known as autosomal recessive Segawa syndrome, has a broad continuous spectrum, manifesting from infancy to young adulthood as abnormal movements in the form of dystonia, rest tremor and Parkinsonism. There are three types of TH deficiency, based on severity of symptoms and responsiveness to L‐Dopa. There is a wide spectrum of the disorder. These are Type (1) TH‐deficient L‐Dopa‐responsive dystonia (the mild form of TH deficiency), (2) TH‐deficient infantile Parkinsonism with motor delay (the severe form), and (3) TH‐deficient progressive infantile encephalopathy.^2^ Up to date, fewer than 100 patients have been published in literature, including one from India.^1^, ^3^, ^4^, ^5^ We present a case of an infant girl with TH‐deficient infantile Parkinsonism and motor delay, with one previously described pathogenic variant and another hitherto unreported large deletion in the *TH* gene.

## 2 CASE REPORT

A 10‐month‐old infant girl, born to nonconsanguineous parents, presented with a history of progressive abnormal movements of body along with a global developmental delay noted since 5 months of age.

Her antenatal period had been uneventful, with normal fetal movements perceived by her mother. She was born via Caesarean section, with birth weight of 3 kg and cried immediately. She was noted to be hypotonic from neonatal period, but did not require admission. At 5‐months of age, she was noted to have rest tremor (Video S1), decreased facial expression, global hypokinesia, and abnormal stiffening of body especially during crying spells. She had attained some personal social milestones like social smile and could make noises, but had significant motor delay. There was no history of seizures. Her anthropometry showed a weight and length below third centile, while head circumference was 42 cm at 50th centile.

A metabolic workup including plasma lactate, ammonia, dried blood spot acyl carnitines, aminoacids, and urinary organic acids—did not show any abnormality, except for mild elevation of lactate. As mild nutritional Vitamin B12 deficiency had been identified, she was treated initially as infantile tremor syndrome with Vitamin B12 supplementation, but this did not show any benefit. Over next few months the hypokinesia, rest tremor and episodic dystonia had worsened. She also started to have prolonged periods of oculogyria, especially in evenings (Video S2). Serum prolactin was elevated (35.76 ng/mL; normal <24 ng/mL). Hence, a biosynthesis defect for catecholamine neurotransmitter was considered, including evaluation of monoamine neurotransmitter metabolites in the cerebrospinal fluid (CSF) and genetic testing of genomic DNA.

CSF studies revealed low levels of homovanillic acid (HVA, 62 nmoL/L; normal range 295‐932 for 0.5‐1 year age group) as well as 3‐methoxy‐4‐hydroxyphenylglycol (MHPG, 9.8 nmoL/L; normal range 51‐112 for 0.5‐1 year age group), while other metabolites were at normal levels (eg, 5‐hydroxyindolacetic acid {HIAA} and 5‐hydroxytryptophan). The CSF HVA:HIAA ratio was 0.3 (normal range 1.5‐3.5). CSF pterins and folates were in the normal range. These findings were compatible with diagnosis of TH deficiency. Based on these findings, the subject was treated, commencing at 11 months of age, with a starting dose of L‐Dopa/Carbidopa (4:1 ratio at 0.5 mg/kg/day) that was gradually increased over months to reach 6 mg/kg/day. After few months, a repeat CSF analysis was performed for monoamine neurotransmitter metabolites, which showed levels of HVA and MHPG in the normal range, but a low folate (5‐methyltetrahydrofolic acid). Folinic acid was started at 1 mg/kg/day and the child showed good in form of significantly improved facial expression with motor and cognitive progress.

On follow up, the child showed a significant motor recovery (Video S3). At 3.5 years of age, she is ambulant including can run and climb stairs without support. Cognitively, she is normal and is performing appropriately for her academic level. The episodic dystonia and oculogyria has subsided completely. She now has normal facial expression. She does have choreiform movements which appear to be related to the prolonged use of L‐Dopa, to which she has responded well on addition of amantadine at dose of 5 mg/kg/day. These movements are not affecting her daily routine function or quality of life (video in pre‐treatment and post‐treatment state provided).

**Figure 1:**
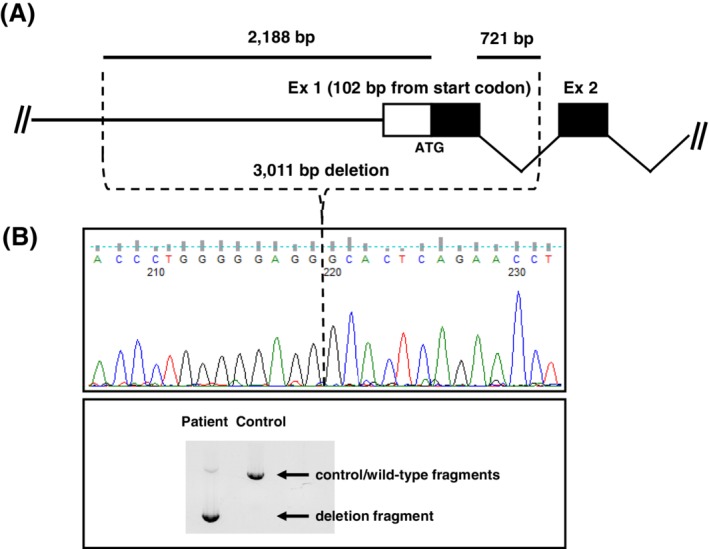
Diagram showing the evidence and extent of large deletion noted in TH gene in heterozygous state. Identification of the novel deletion allele c.‐2188_103‐194del in the TH gene that extends over 3,011 bp and includes the entire exon 1. A, Schematic of the deletion showing the 5′ portion of the human TH gene, including exons 1 and 2. B, Lower part: Agarose gel‐separation of long‐range PCR products using flanking primers (not shown). Upper part: result of Sanger sequencing of the “deletion fragment,” revealing the molecular details of the DNA‐breakpoint (RefSeq NG_008128.1)

*Molecular testing* was performed by next generation sequencing using an Illumina MiSeq device to sequence the coding exons and flanking introns from a defined panel of genes, including the *TH* gene (reference sequence ENSG00000180176, NM_199292.2; subpanel “infantile parkinson dystonia”; more details can be obtained by contacting the authors). This analysis revealed the following variants in the *TH* gene. One heterozygous single nucleotide missense variant, previously reported as likely pathogenic in exon 11 (c.1196C>T; p.Thr399Met) (http://www.ncbi.nlm.nih.gov/clinvar),^6^ and another homozygous variant in exon 1 (c.16G>A; p.Ala6Thr) which was likely not to be pathogenic. The variants were further tested in parents using the Sanger sequencing method. It was noted that while both the variants were present in heterozygous form in the mother, none of these variants were present in the father. This was interpreted that both variants were inherited in *cis*‐form from the mother, and the father was suspected to be harboring a deletion involving exon 1, since the exon 1 variant was observed in apparent homozygous form in the child. Further analyses, including long‐rang PCR, revealed a novel deletion involving exon 1, c.‐2188_103‐194del that was present in a heterozygous state in the child's DNA (Figure 1). Furthermore, the father of child was noted to carry the same variant in heterozygous state. Thus, the detection of these two compound heterozygote variants in the *TH* gene, c.1196C>T;p.Thr399Met and 3011 base pair deletion (c.‐2188_103‐194del) confirmed the diagnosis of TH deficiency.

## 3 DISCUSSION

TH deficiency is a treatable disorder, if recognized early in the disease course. Because of the rarity, the awareness of this condition is less especially amongst Pediatricians and general physicians. The present case was initially thought to be a vitamin B12‐deficiency related infantile tremor syndrome, but was further investigated after no response to vitamin B12 was noted. The infantile tremor syndrome is a well‐known entity in the Indian scenario, even today.^7^ The present case was confirmed to have TH deficiency only after definitive neurotransmitter analysis and genetic testing. Our case showed clinical presentation consistent with type 2 TH deficiency, with severe infantile Parkinsonism‐like symptoms. She demonstrated a good clinical improvement upon therapy following a timely diagnosis. A secondary cerebral folate deficiency was treated leading to better prognosis. There is evidence in another neurotransmitter disorder (AADC deficiency) that secondary cerebral folate deficiency can develop with L‐dopa therapy—O‐methylation of the excessive amounts of L‐Dopa to 3‐OMD depletes methyl donors including SAM and 5‐MTHF.^8^

Severe infantile Parkinsonism type of TH deficiency has been scarcely reported with fewer than 100 cases reported till date.^2^ In one study from India, two siblings were described presenting similar to our case and showing improvement upon early treatment.^3^ The molecular studies in the two infants revealed compound heterozygous variants, one of them being a common, p.Arg233His in exon 6 of TH gene. Our patient harbored another previously reported variant, p.Tyr399Met along with a novel large deletion encompassing exon 1 in TH gene. The variant, p.Tyr399Met has been studied previously in a 16‐week‐old human fetus revealing a decreased expression in TH and in dopaminergic proteins in the fetal brain.^9^ This variant was described in the sibling of the fetus who presented at 3 years of age and showed a severe phenotype at 17 years of age. The second variant in our case was a novel large deletion and is only the second report of a large deletion in TH gene. The previous deletion reported spanned from intron 11 to the 3′end in exon 13, encompassing a segment of 716 bp (c.1197 + 25_1391del) including exon 12 and part of exon 13.^10^ The deletion observed in our case involved region of TH gene 5′ of exon 1 up to a part of exon 1(c.‐2188_103‐194del). There is another report of a novel deletion of entire *TH* gene in an adult with Parkinson disease.^11^ This was a heterozygous whole gene deletion in a 59‐year‐old woman who presented with symptoms of PD at age 54 years, and who was responsive to L‐Dopa. A genotype phenotype correlation is difficult to establish as all cases with a deletion, including ours, have been in heterozygous states, observed either alone or along with a different point mutation. In our case, the reported variant is associated with a severe phenotype.^9^

The case presented highlights the importance of keen clinical observation, appropriate and timely biochemical investigations (CSF neurotransmitters, supported by a high prolactin level) to achieve an accurate diagnosis. Genetic testing can be challenging, as a large deletion was not expected and was detected ingeniously using innovative molecular methods. A timely initiation of appropriate therapy has led to a good outcome in the otherwise severe disorder.

In conclusion, it is appropriate to mention that in cases with negative results on Next Generation Sequencing technique, or if only one heterozygous variant is found in TH gene and if clinical suspicion is high, deletion analysis by other molecular or cytogenetic techniques is warranted.

## CONFLICT OF INTEREST

All the authors have no competing interest.

## ETHICAL APPROVAL STATEMENT

No ethical approval was required as the testing in child was performed according to medical indication, as part of standard of care.

## PATIENT CONSENT

An informed consent has been taken from father of the child, for publication as well as for sharing video of the child. Documentation of approval from the Institutional Committee for Care and Use of Laboratory Animals (or comparable committee)—Not applicable.

## AUTHOR CONTRIBUTIONS

S.B.M. wrote the manuscript and was involved in organizing biochemical and genetic testing. V.J. managed the case and provided crucial inputs in the writing of manuscript. B.T. performed biochemical (CSF neurotransmitter analysis) as well as genetic analysis in the patient, in addition to providing useful inputs for writing of manuscript.

## Supporting information


**Video S1** Pretreatment early infancy

**Video S2** Pretreatment oculogyria

**Video S3** At 3.5 years age, post treatment
